# Influence of Lateral Movement on Level Behavior of Adhesion Force Measured Repeatedly by an Atomic Force Microscope (AFM) Colloid Probe in Dry Conditions

**DOI:** 10.3390/ma14020370

**Published:** 2021-01-13

**Authors:** Ping Li, Tianmao Lai

**Affiliations:** School of Mechanical and Electric Engineering, Guangzhou University, Guangzhou 510006, China; leeping@gzhu.edu.cn

**Keywords:** pull-off force, force-displacement curve, sliding velocity, level behavior, atomic force microscope

## Abstract

An atomic force microscope (AFM) was operated to repeatedly measure the adhesion forces between a polystyrene colloid probe and a gold film, with and without lateral movement in dry conditions. Experimental results show that the adhesion force shows a level behavior without lateral movement and with a small scan distance: the data points are grouped into several levels, and the adhesion force jumps between different levels frequently. This was attributed to the fact that when the cantilever pulls off the sample, the contact area of the sample is not exactly the same between successive contacts and jumps randomly from one to another. Both lateral velocity and material wear have little influence on level behavior. However, with a medium scan distance, level behavior is observed only for some measurements, and adhesion forces are randomly distributed for the other measurements. With a large scan distance, adhesion forces are randomly distributed for all measurements. This was attributed to the fact that the cantilever pulls off the sample in many different contact areas on the scanning path for large distances. These results may help understand the influence of lateral movement and imply the contribution of asperities to adhesion force.

## 1. Introduction

Adhesion between materials is becoming increasingly important. Since the adhesion force is the dominant factor in the failure of micro-electromechanical systems (MEMS) on the grounds of fabrication and use [1], it is increasingly significant with the rapid development of MEMS. As a representative MEMS device, micro-switches have been used in wide applications, from industrial instrumentation, smart antennas, phase shifters to cell phones [2,3]. Failure of most micro-switches is generally caused by increasing adhesion force during repeated make-and-break contacts [4,5]. Therefore, the adhesion behavior for repeated contacts on a micro- and nanoscale is keenly demanded to develop a sophisticated understanding. On the other hand, adhesion force may involve several different mechanisms on a microscale, nanoscale and molecular scale. It is also important to understand the evolution rule of adhesion force with time, to better control the adhesion force.

In recent years, atomic force microscope (AFM) has become a problem-solving instrument for long-range and short-range forces between two surfaces. It can measure the adhesion force from microscale down to molecular level under different kinds of environments by recording a force-displacement curve with high spatial resolution. Repeated contact between an AFM tip and a substrate can be used to simulate the closing and opening of the microcontacts of micro-switches [6,7]. Additionally, a colloid probe (formed by attaching a microsphere to the end of a cantilever) is usually used to prevent severe wear and tear of the material [8].

During the measurement of adhesion forces in AFMs, deflection of a probe is detected when it touches a sample and retracts back. During this process, the influence of lateral movement can be investigated with linear or circular displacement [9]. The influence of lateral movement in a nanometer-sized contact in air has been investigated in some studies. Noël et al. [10] reported that van der Waals (vdW) force remains constant, and capillary force decreases logarithmically with lateral velocity in a range between 5.652 μm/s and 565.2 μm/s. However, Sirghi [11] proposed that adhesion force first decreases steadily and then remains unchanged with lateral velocity after 3 μm/s for a nanometer-sized contact. The decrease is usually attributed to the disappearance of capillary force [12]. On a microscale, a decrease has also been reported and was attributed to the breakage of adhesive junctions [13]. Recently, Lai et al. [14,15] found that with the increase of lateral velocity, adhesion force decreases logarithmically at large velocities in air. This decrease was attributed to the increase in size of water bridges with contact time formed in the contact zone.

Some studies have focused on the behavior of adhesion forces at a single location by repeated contact. However, in these studies, consecutive measurements were performed with a relatively small measurement number of times to examine the reproducibility: 15 times [16], 20 times [17], 50 times [18], and 100 times [19]. In this research, the reported data shows no characteristic trend. However, it was reported that an increasing trend in measurement number of times can be observed, provided that plastic deformation of asperities occurs [20]. Furthermore, in very dry conditions, trends can increase, decrease or remain unchanged because of electric charging [21]. If the adhesion force is contact-time dependent (increasing logarithmically), data with repeated contacts will show a sharp increase at first, then a slow increase, and finally remain constant [22,23]. Recently, Lai et al. [24] put forward that the measured data points in a very dry environment are grouped into several levels, and frequent jumping behavior between different levels was observed. This “level behavior” was attributed to different sets of asperities on a sample surface because of non-linear factors. Intuitively, if the sample is forced to move laterally with a very small scan distance, the level behavior will remain unchanged since the asperities set is almost the same. However, one might wonder what will happen when the scan distance is large. Then, several questions can be raised. (1) Will the level behavior become unchanged at a large scan distance or not? (2) If the level behavior becomes unclear, are the adhesion forces randomly distributed or not? (3) If the adhesion forces are randomly distributed at a very large scan distance, does a critical scan distance exist or not? (4) With lateral movement, will lateral velocity influence level behavior or not? Therefore, adhesion behavior determined by repeated contact with and without lateral movement demands clarification, and further study is needed.

In this paper, adhesion force data have been collected repeatedly on an Au film surface with and without lateral movement by using a microsphere probe in very dry conditions. The AFM piezo with a sample can be driven to move laterally (in a to and fro motion), with different scan distances and scan rates. For each set of parameters, 512 force curves were collected to extract the adhesion forces. The evolution of the data with repeated contacts was studied, and adhesion force behaviors under different parameters were analyzed with mutual comparisons. The behaviors with and without lateral movement were discussed. Different behaviors were attributed to different sets of asperities on the substrate, which can be contacted by the probe. The aim is not only to demonstrate the influence of lateral movement on level behavior but also to imply the contribution of asperities to the magnitude of adhesion force.

## 2. Materials and Methods

### 2.1. Sample Preparations and Characterization

**Figure 1:**
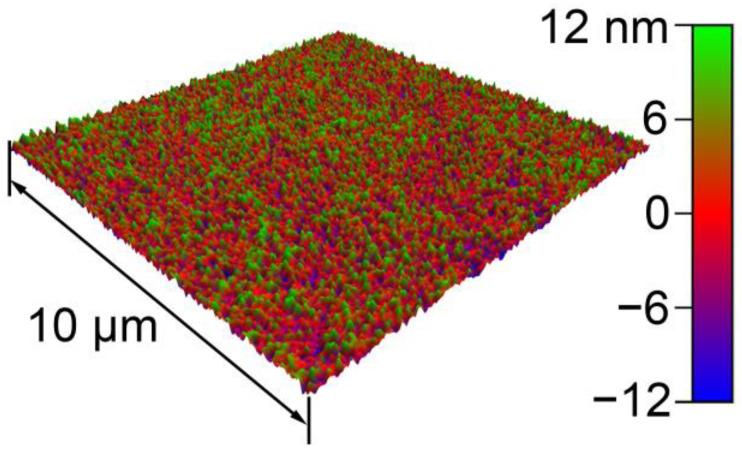
Topographic image of the sample scanned on an AFM (scan size = 10 μm × 10 μm).

In the experiments, an Au film surface was used as the sample, manufactured by magnetron sputtering physical vapor deposition (MSPVD). The substrate was an N-type silicon wafer. The bottom layer of the film was Cr with a thickness of ~20 nm, and the upper layer was Au with a thickness of ~1200 nm. The Au film was used because of its chemical inertness during contact measurements. The image of the sample topography is shown in Figure 1. This image was obtained with the tapping mode of an AFM (MFP-3D Classic, Asylum Research, Santa Barbara, CA, USA). Based on the image, the root-mean-square roughness was 5.693 nm. Before the measurements, the sample was cleaned ultrasonically, first in ethyl alcohol (10 min) and then in deionized water (10 min).

### 2.2. Measurement Methods for Adhesion Forces

An AFM (Being Nano-Instruments CSPM-4000, Guangzhou, China) was used to collect adhesion force data. The AFM was placed in a glove box (Lab2000, Etelux Inert Gas, Beijing, China) to adjust the atmosphere around it. The box was filled with nitrogen in high purity, where the water content was below 0.5 ppm (temperature = 28 ± 1 °C). 

**Figure 2:**
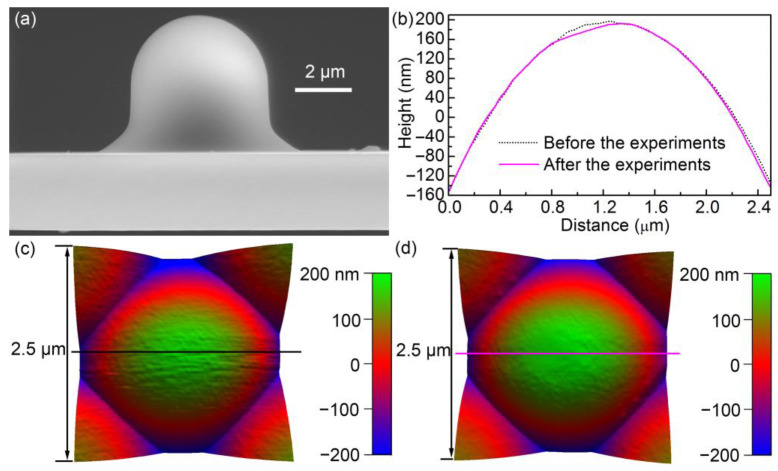
(**a**) SEM image of the probe before the experiments. (**b**) Cross-sectional profiles before and after the experiments, created by the straight lines shown in (**c**,**d**). (**c**,**d**) 3D AFM images of the microsphere before and after the measurements, respectively. Scan size = 2.5 × 2.5 μm.

The data were collected with a colloidal probe during the experiments. It was fabricated by adding a polystyrene (PS) microsphere to a tipless cantilever (TL-CONT, Nanosensors, Neuchatel, Switzerland) with glue. The microsphere (diameter = ~4.8 μm) was purchased from Nano-Micro Tech, Suzhou, China. Images of the microsphere obtained in a scanning electron microscope (SEM) and an AFM are shown in Figure 2. SEM images were collected with a commercial SEM (Quanta 200 FEG, FEI Company, Eindhoven, The Netherlands). Topographic AFM images were obtained by an inverse imaging method with a grating (TGT01, NT-MDT, Moscow, Russia) with the MFP-3D AFM. The wear and tear of the microsphere can be seen from the image after the measurements. During the experiments, the probe was tilted by ~17° in the CSPM-4000 AFM. Therefore, the wear scar (with diameter of ~0.55 μm) was not just the peak of the microsphere. The normal spring constant of the probe (0.452 N/m) was measured using a thermal method on the MFP-3D AFM [25].

**Figure 3:**
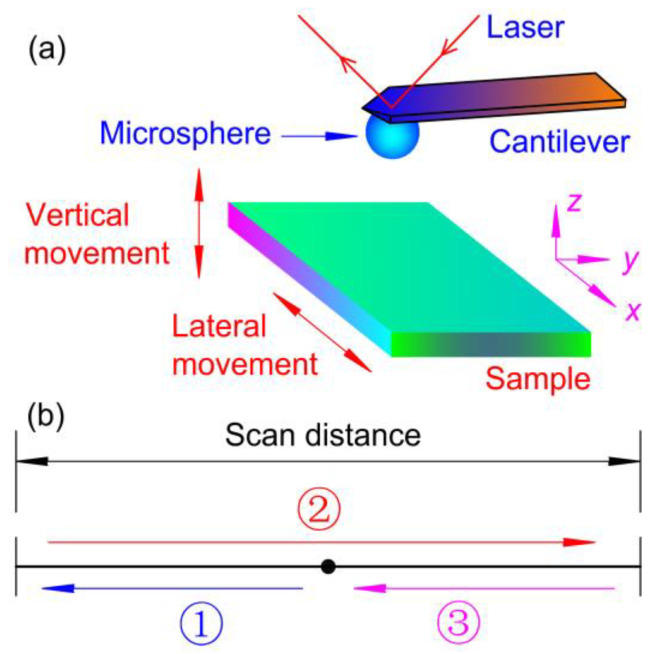
(**a**) Schematic of the experimental set-up for collected force curves. Two simultaneous motions (vertical and lateral) are shown. (**b**) The scanning cyclic process of the sample. Arrows and numbers with circles are used to show a cycle.

Adhesion force is determined by obtaining a force curve. In the CSPM-4000 AFM, a probe is fixed in an immobile probe holder, and a substrate can move in three directions. In order to study the influence of lateral movement on the behavior of adhesion force, a sample can be moved laterally in a direction perpendicular to the cantilever without interruption during the collection of force curves. The experimental set-up and the scanning process of a cycle are shown in Figure 3. Before scanning, the sample is in the middle of a scanning distance. As shown in Figure 3b, for one cycle, the sample moves following the program: to left first, then to right, and at last to the left again. The sample can be moved with different scan distances (0 ~ 80 μm) and different scan rates (0.001~100 Hz). The lateral velocity ($V_{lateral}$) can be calculated as

(1) $$V_{lateral}=2f_{scan}d$$

where $f_{scan}$ is the scan rate, *d* is the scan distance.

**Figure 4:**
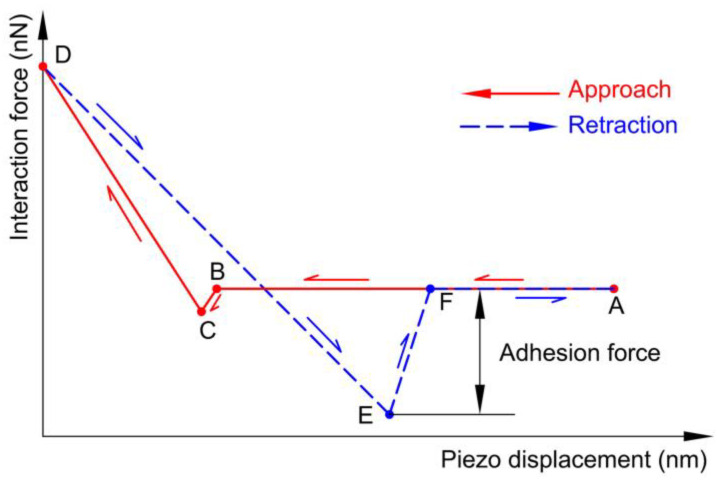
Schematic diagram of a force curve.

A force curve is recorded by monitoring the cantilever deflection with the approach and retraction of a substrate (moving up and down). The schematic diagram of the force curve is shown in Figure 4. When the distance between the microsphere and the substrate is large (A), the interaction between them vanishes. With the upward movement of the substrate, the cantilever may bend downward due to attractive forces. If the distance between the surfaces is small enough, the microsphere jumps into contact with the substrate (B,C). The microsphere is pressed upward with the bend of the cantilever as the substrate goes up. The substrate stops at one point with a maximum load (D). During the retraction, the interaction between these surfaces decreases gradually. After some time, the cantilever bends downward. However, they are still in touch due to the adhesion force. The deflection of the cantilever becomes larger and larger, until it jumps back to its original point (E,F). Forces were extracted from the force curves. During the measurements, the maximum force = ~50 nN, Z-piezo velocity = ~14.6 μm/s, and dwell time = 0 s. For each selected set of parameters, 512 force curves were collected.

## 3. Results

**Figure 5:**
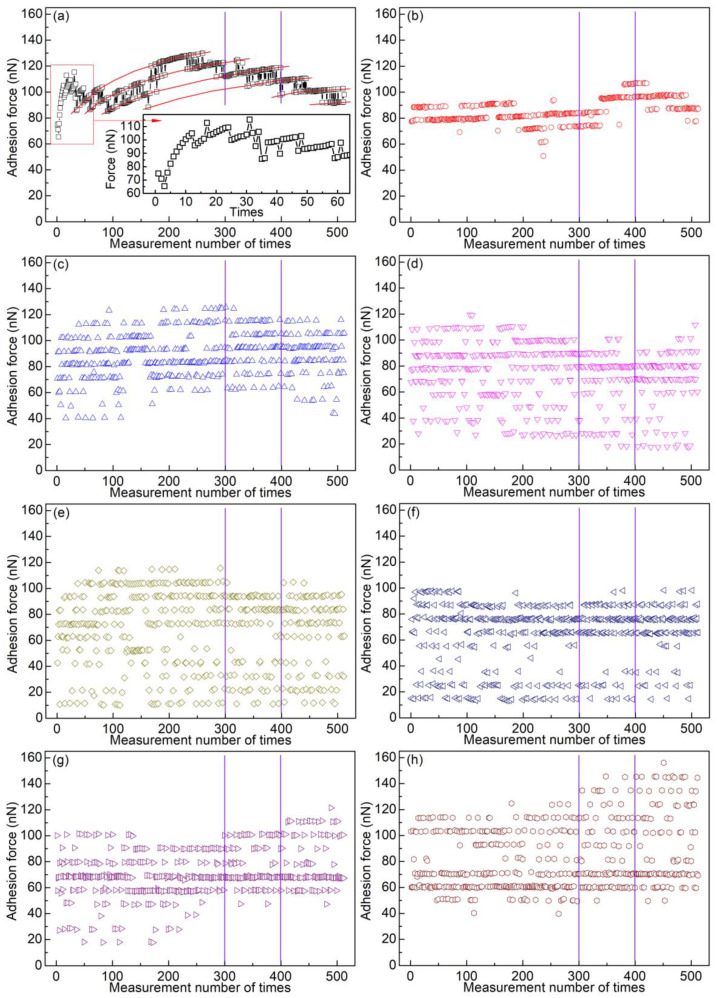

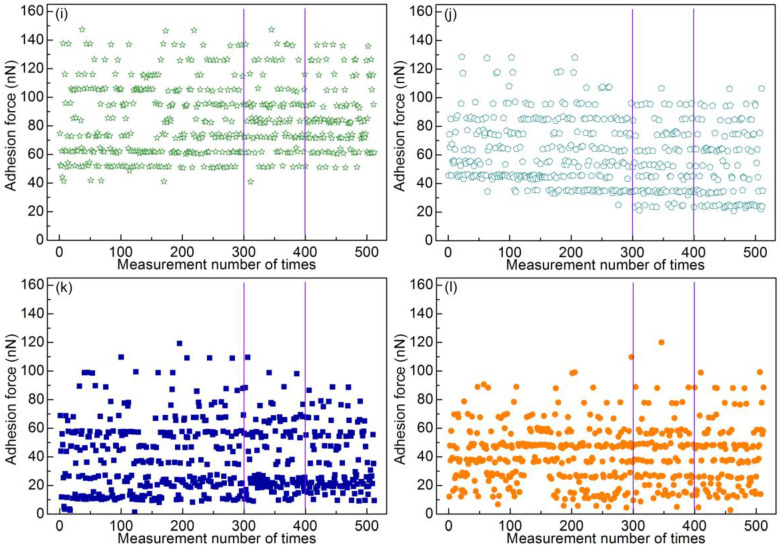
Adhesion force as a function of measurement number with a scan rate 1 Hz and different scan distances: (**a**) 0, (**b**) 0, (**c**) 10 nm, (**d**) 20 nm, (**e**) 40 nm, (**f**) 80 nm, (**g**) 160 nm, (**h**) 320 nm, (**i**) 640 nm, (**j**) 1 μm, (**k**) 2 μm, and (**l**) 4 μm. Two segments (100 data points) are marked by vertical lines, which are selected to be replotted in the same figure. The inset in (**a**) shows the segment’s enlarged view in the corresponding box, and the polylines in (**a**) are used in order to guide the eye. In (**a**), straight lines are displayed between data points to indicate jumping behavior between different levels. In (**b**–**l**), there are no lines between data points for the sake of clarity.

In dry nitrogen, a location (Location 1) on the substrate was randomly chosen to collect data consecutively. At first, adhesion forces were measured 512 times at Location 1, without lateral movement of the sample. This process was repeated once again to check the reproducibility of the experiments. Then, the adhesion force was measured consecutively with the lateral movement of the sample at different scan distances (Location 1 was the middle position of a scan distance). The scan rate was set as 1 Hz, and the scan distance was between 10 nm and 4 μm. For each scan distance, 512 force curves were recorded consecutively. Figure 5 shows the obtained data points.

In Figure 5a–j, the measured points are grouped into different levels, and the adhesion force jumps in a certain regularity between different levels. When measuring adhesion forces consecutively at one location of a sample, if the real contact area is not exactly the same among these successive contacts, the data points are usually grouped into several levels. This phenomenon is referred as the “level behavior” of adhesion force [24]. In Figure 5a, data points in the same level are connected by polylines. The adhesion force decreases for the first three data points, and then increases sharply. Likewise, the adhesion force increases largely for one level at first and then slightly with the measurement number of times. In Figure 5b, it is seen that the adhesion force for one level increases slightly. Nevertheless, the adhesion force for one level remains almost the same (Figure 5c–j). The adhesion force fluctuates modestly for one level when the scan distance is between 0 and 640 nm. The fluctuations become larger when the scan distance is 1 μm. Here, the fluctuations are evaluated by the difference of two sequential points. When the scan distance is between 10 nm and 1 μm, the differences in the magnitude of force between two adjacent levels are basically the same for one scan distance. However, when the scan distances are 2 and 4 μm, most data points are randomly distributed, although level behavior can also be observed for some data points, as shown in Figure 5k,l. The adhesion forces are all lower than 160 nN.

**Figure 6:**
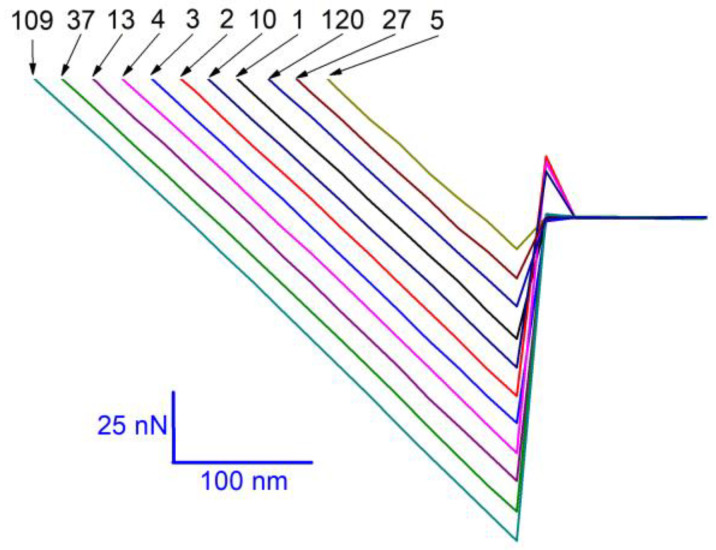
Several typical force curves of retraction segments (11 lines) obtained with a scan rate of 1 Hz and a scan distance of 40 nm. The numbers are used to show the measurement numbers of these force curves.

**Figure 7:**
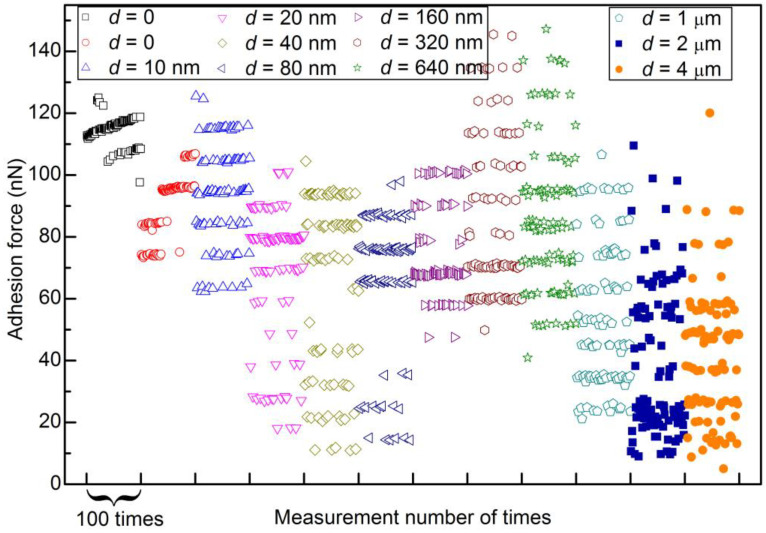
Adhesion force as a function of measurement number with a scan rate of 1 Hz and different scan distances. For comparison, a segment of 100 data points for each scan distance is selected to be replotted here from a figure of 512 data points with the same measurement parameters (from Figure 5).

Figure 6 shows several typical force curves of retraction segments, which correspond to some of the data points shown in Figure 5e. These force curves are selected from different levels and used to demonstrate the level behavior more clearly. For comparison, a segment of 100 data points for each scan distance is selected (marked by two vertical lines in Figure 5). These segments are replotted in Figure 7. The level behavior of adhesion force at different scan distances can be clearly seen.

**Figure 8:**
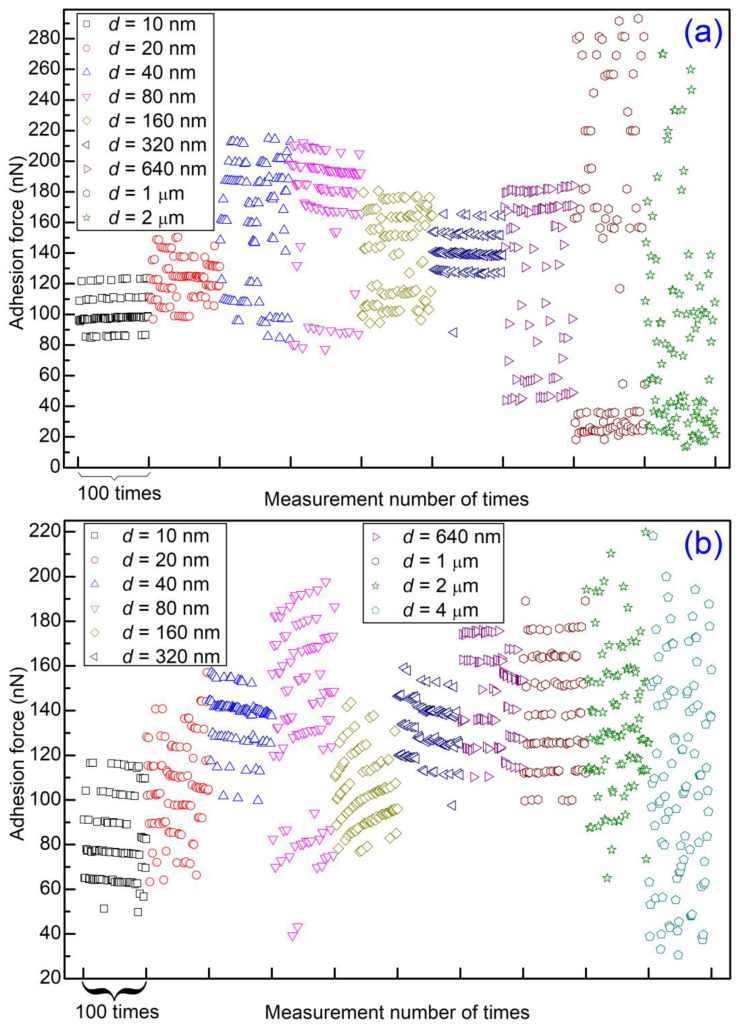
Adhesion force as a function of measurement number with a scan rate 1 Hz and different scan distances. For comparison, a segment of 100 data points for each scan distance is selected to be replotted here from a figure of 512 data points with the same measurement parameters: (**a**) from Supplementary Figure S1 and (**b**) from Supplementary Figure S2.

With the increase of the contact number, the microsphere wears gradually. One may suspect that the randomly distributed data points obtained when the scan distance was 2 or 4 μm are due to the microsphere’s wear and tear. In order to rule out such a possibility and check the reproducibility of the experiments, two more sets of experiments were carried out with almost the same experiment parameters. The adhesion forces obtained are given in Supplementary Figures S1 and S2. Also, a segment of 100 data points for each scan distance is selected, and these segments are replotted in Figure 8. Once again, level behavior can be observed clearly for small scan distances but not for large scan distances (>1 μm). However, different trends (increasing, decreasing and unchanged) were observed for one level. Even more, the adhesion force for one level sometimes jumps slightly higher or lower with discontinuity. The maximum adhesion force of these two sets of experiments is as large as ~280 nN, which is much larger than that of the previous set of experiments (<160 nN). The wear and tear of the materials may contribute to the small jump and larger magnitudes. 

**Figure 9:**
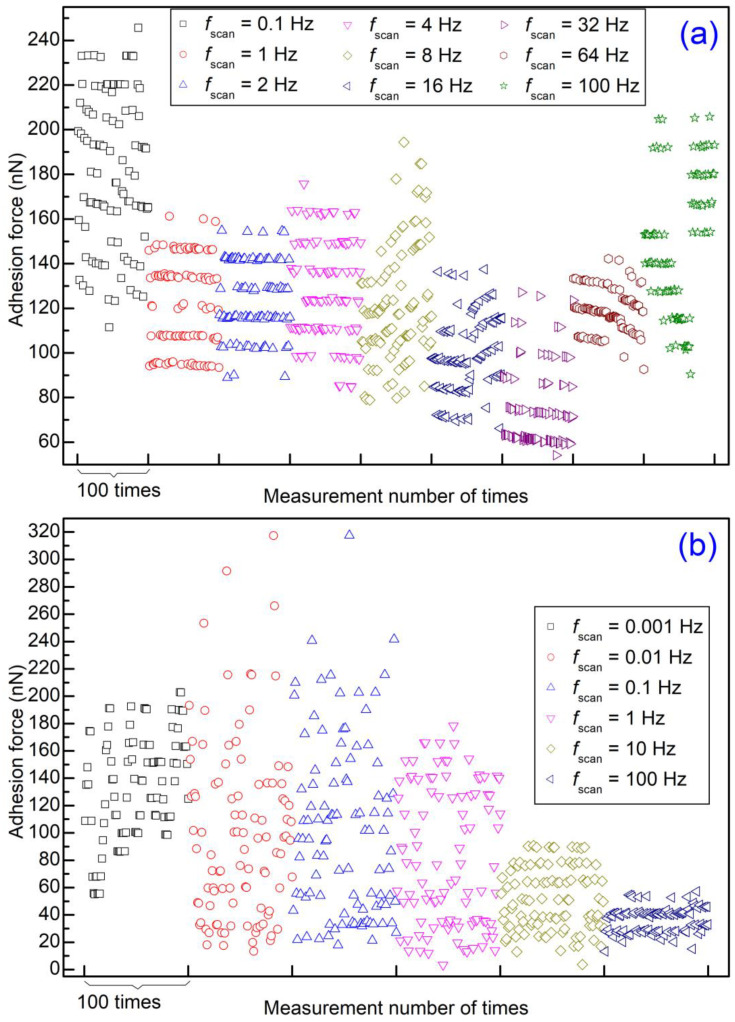
Adhesion force as a function of measurement number at different scan rates: (**a**) with a scan distance 640 nm, from Supplementary Figure S3; (**b**) with a scan distance 6.4 μm, from Supplementary Figure S4. For comparison, a segment of 100 data points for each scan distance is selected to be replotted here from a figure of 512 data points with the same measurement parameters.

To study the effect of lateral velocity on level behavior, the adhesion force was then measured consecutively with a scan distance of 640 nm or 6.4 μm at different scan rates. For d = 640 nm, by setting different scan rates, the lateral velocity was between 128 nm/s and 128 μm/s. Meanwhile, the lateral velocity was between 12.8 nm/s and 1280 μm/s for d = 6.4 μm. For each scan rate, 512 force-displacement curves were recorded consecutively. The obtained adhesion forces are shown in Supplementary Figures S3 and S4. Also, a segment of 100 data points for each scan distance is selected, and these segments are replotted in Figure 9. The outcome shows that level behavior can be observed using d = 640 nm at different scan rates but not d = 6.4 μm. It seems that lateral velocity has little effect on level behavior.

**Figure 10:**
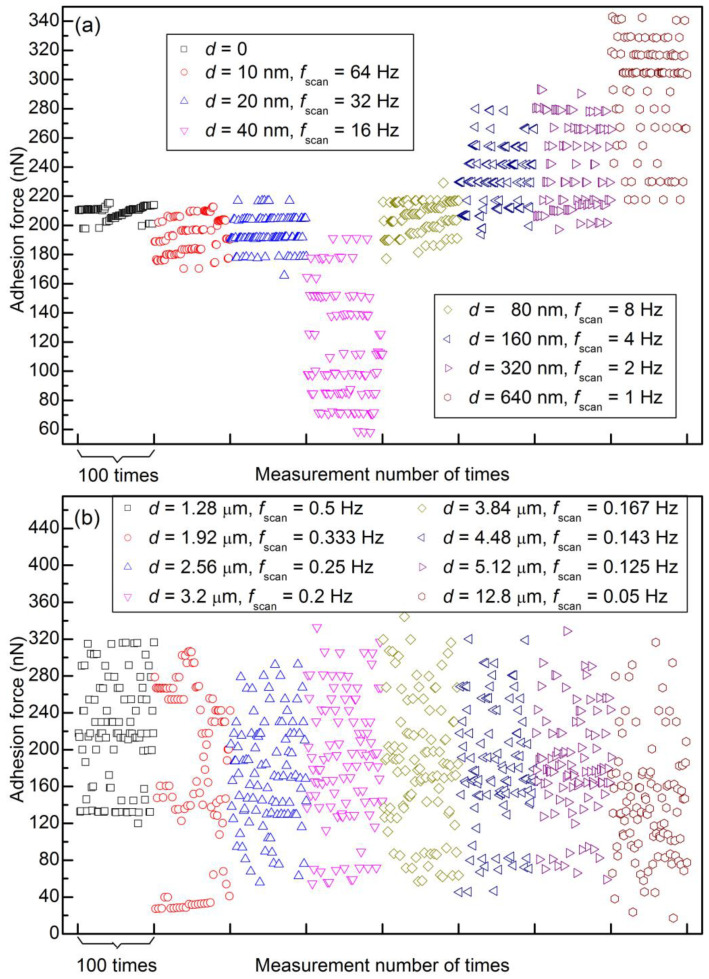
Adhesion force as a function of measurement number with different scan distances and rates (the lateral velocity is 1.28 μm/s except for the first segment): (**a**) scan distance <1 μm and (**b**) scan distance >1 μm. For comparison, a segment of 100 data points for each scan distance is selected to be replotted here from a figure of 512 data points with the same measurement parameters (from Supplementary Figure S5).

To eliminate the lateral velocity effect, the lateral velocity was kept constant in the next set of experiments (1.28 μm/s). This time, the adhesion force was measured consecutively, first without lateral movement and then with the same lateral velocity (by adjusting different scan distances and different scan rates). The adhesion forces are shown in Supplementary Figure S5, and selected segments of 100 data points are shown in Figure 10. Level behavior can be observed clearly when the scan distance is less than 1 μm but not when the scan distance is larger than 1 μm. With the increase in scan distance, there are three stages with different distribution behavior: (1) almost all of the data points are in some levels (0~640 nm for this set of experiments); (2) some data points are in some levels, while the others are not (640 nm~1.92 μm); (3) almost all of the data points are randomly distributed (1.92 μm~12.8 μm). The maximum adhesion force of this set of experiments is as large as ~460 nN (see Supplementary Figure S5h), which may be the result of material wear and tear.

## 4. Discussion

Generally, adhesion force has different contributions: capillary force, vdW force, and electrostatic force [26]. In dry conditions, the capillary force vanishes, and the latter two are dominant. Furthermore, the electrostatic force may be much larger than the vdW force if contact electrification (CE) occurs. In the CE theory, when two surfaces in contact are separated, one has a positive net charge and the other has a negative net charge [27,28]. In a “triboelectric series”, different materials are arranged to predict surface polarity after CE [29,30,31]. When a PS microsphere comes into contact with Au film, it has been reported that the PS microsphere will be negatively charged and the Au film will be positively charged [31].

**Figure 11:**
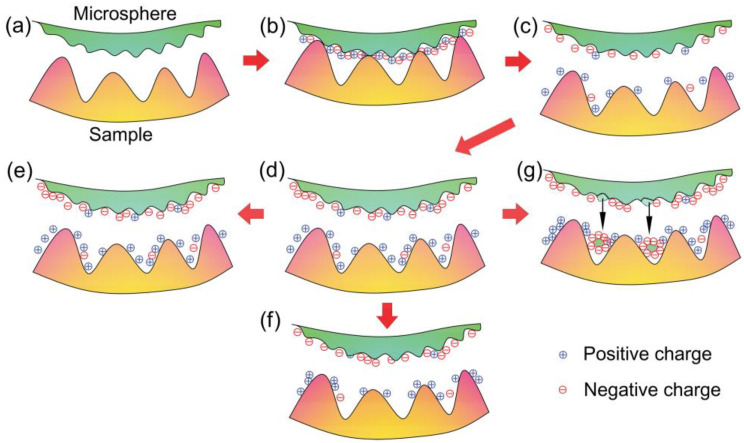
Schematics of the interfacial situations with charge distributions between the microsphere and Au film. (**a**) At the beginning, there is no net charge on either surface (neutral). (**b**) At first contact, an electrical double layer is formed across the interface. (**c**) After separation, the microsphere surface has negative net charges, and Au film has positive net charges. (**d**) Charges on both surfaces are accumulated through repeated contact. That is, the charge density increases gradually. (**e**) There is an upper limit to the amount of charge contained on one surface, leading to a nearly unchanged charge density. (**f**) The charges can be dissipated during repeated contact, resulting in decreasing density. (**g**) Net charge density decreases, since torn-off patches of material on the microsphere surface are transferred to the opposite surface (material transfer).

Figure 11a–c schematically show the process of CE in the interface between the microsphere and Au film. Initially, there is no net charge on both surfaces without contact, as shown in Figure 11a. When the microsphere is pressed on the sample surface, the transfer of charges occurs across the interface, until it is saturated. In this way, an electrical double layer is formed in the contact zone (Figure 11b). After separation, the PS microsphere will be negatively net charged and the Au film will be positively net charged (Figure 11c). It should be noted that, there are positively and negatively charged regions on a small scale on each surface. Terris et al. [32] reported that this phenomenon can happen on a microscale. Furthermore, Baytekin et al. [33] suggested that non-uniformity can also be found on a nanoscale.

After separation, an electric field is built up, resulting in an electrostatic force. It is assumed that contact between the microsphere and Au film can be viewed as a parallel plate capacitor, and the charge densities on both surfaces are the same. Then, the electrostatic interaction can be expressed as [34,35,36]

(2) $$F_{el}=\frac{A\sigma^{2}}{2\varepsilon_{0}}$$

where *A* is the actual contact area, *σ* is the charge density, and *ε*_0_ is the permittivity of the vacuum.

Based on the theory of CE, surface charge density tends to increase gradually after consecutive contact in one location (Figure 11d). There are some reasons for the accumulation of charges. Firstly, the material of the microsphere is insulated. Therefore, trapped surface charges on the microsphere surface cannot leak effectively. Secondly, the transfer of charges into the microsphere body is also difficult [37,38], although charge on Au film may dissipate gradually. Lastly, charge dissipation to gas is difficult in dry conditions (water content < 0.5 ppm). Therefore, charge density increases gradually with repeated contact. From Equation (2), *F*_el_ will increase gradually due to an increased charge density, provided that the contact area is constant. With the increase in charge density, the electric field strength between two surfaces increases. It should be noted that the electric field between these surfaces must repel for the charge to transfer [39]. Thus, charge transfer in one direction becomes increasingly difficult with time. At some point, the accumulation of charges stops, leading to a nearly unchanged charge density. That is to say, there is an upper limit to the amount of charge (Figure 11e). According to the above reasons, the evolution of adhesion force can be described as follows: increasing sharply, then increasing slowly, then becoming stable or changing slightly.

As well as an increasing trend, a decreasing trend in adhesion force for one level can be observed. The decreasing trend was attributed to the decrease in charge density. Firstly, neutralization of charges can occur to some extent with repeated contact. Secondly, charge leakage is inevitable on both the conductive Au film and the insulated microsphere surface, although the effect may be small on the insulated surface. Therefore, charges on the surfaces will dissipate to some extent (Figure 11f). Finally, the density of net charge decreases, provided that torn-off patches of material (due to wear) on the microsphere surface are transferred to the opposite surface (Figure 11g). Baytekin et al. [40] reported that the effect of material transfer can be large, and the charge polarity of one surface can even be reversed. The effect of material transfer cannot be neglected because of the worn microsphere after the experiments (Figure 2).

**Figure 12:**
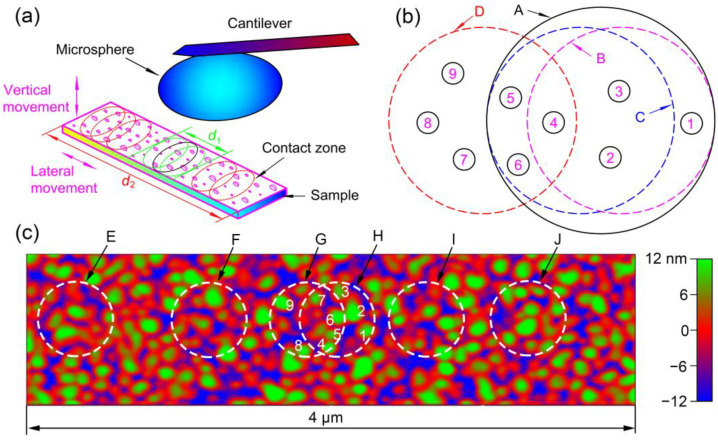
(**a**) Schematic of force curve measurement with two scan distances and contact zones shown on the sample surface. There are lots of asperities on the sample surface. (**b**) Contact areas on Au film for the microsphere-sample contact pair. Without lateral movement, Circle A is the maximum area that can be touched by the microsphere, and Circles B–C are the actual areas for a single contact. Circle D is an actual contact area with lateral movement. Numbers 1–9 with circles are the asperities on Au film. These asperities are under a certain load when the microsphere is in contact with the sample. (**c**) Contact areas in a scanning area with scan size 4 μm × 1 μm, which was randomly chosen on the Au film surface. Circles E–J are the actual areas for a single contact. The diameters of these circles are the same (~500 nm). Numbers 1–9 are used to mark 9 different asperities, which can be touched by the microsphere when the contact areas are Circles G and H.

In terms of lateral movements of the sample during force curve collection, the pull-off areas (when the microsphere jumps off Au film, also referred to as “contact areas”) on the sample surface are undecided due to the random nature of a pull-off process. That is, the contact area can be any area on the scanning path, and the contact area for a certain force curve is special. The contact scenario between the microsphere and Au film is shown in Figure 12a. There are lots of asperities with different sizes on the sample surface. There may be many asperities in a contact area. However, only higher asperities are under a certain load when the microsphere is in contact with the sample. When the scan distance is small (d1), the contact areas overlap with each other to some degree. When the scan distance is large (d2), the contact areas may be very far from each other. Even without lateral movement of the sample, the real contact area may be slightly different between successive contacts. This was attributed to the fact that the vertical movement of the sample (drove by the Z-piezo) cannot be entirely linear [24]. Thus, the asperities on Au film may be different for consecutive contacts, even without lateral movement of the sample.

In Figure 12b, Circles B and C represent actual contact areas. Circle A is the maximum area that can be touched by the microsphere without lateral movement of the Au film. Since the charges are distributed around asperities, the electrostatic force will suddenly increase or decrease if the contact area jumps from one to another, depending on the number of asperities and the charge distribution in both areas. In Figure 12b, Circles B and C have three asperities in common. When the contact area is Circle B, the adhesion force (*F*_B_) is the summation of the contribution of Asperity 1 (*F*_1_) and Asperities 2–4 (*F*_2–4_). When the contact area is Circle C, the adhesion force (*F*_C_) is the summation of the contribution of Asperities 5–6 (*F*_5–6_) and Asperities 2–4 (*F*_2–4_). Here, the contributions of lower asperities are neglected for the sake of statement convenience. If, at first, the contact area is Circle B and then it jumps to Circle C, the adhesion force will increase from *F*_B_ to *F*_C_ (increasing by Δ*F* = *F*_5–6_ − *F*_1_). If the contact area jumps back to Circle B, the adhesion force also jumps from *F*_C_ back to *F*_B_, provided that all the conditions remain the same (for example, all the charges around the asperities remain unchanged, and no asperity wears or is damaged). In a similar way, if, at first, the contact area is Circle C and then it jumps to Circle D, the adhesion force will also jump to another level. The adhesion force difference between the two levels is the difference between the contribution of Asperities 7–9 and Asperities 2 and 3. In this way, the measured data points are grouped into different levels, both without lateral movement and when the scan distance is small. 

From the discussion above, a level is corresponding to some higher asperities on Au film, which come into contact with the microsphere under a normal load. When the scan distance is small, the number of asperities on the scan path is limited. This is the reason why level behavior still exists when lateral movement of small distances is applied. For example, in Figure 12c, the diameters of the contact areas (circles) are assumed to be ~500 nm. The distance between Circles G and H is ~200 nm. If the scan distance is ~200 nm and Circles G and H are two extreme positions, there are only 9 asperities in the scanning path that can be touched by the microsphere. Therefore, there are only several sets of asperities on the sample surface that can be touched by the microsphere, ultimately resulting in several levels. Usually, the number of levels is small without lateral movement and increases when lateral movement is applied, since there are more asperities that can be touched by the microsphere. Moreover, the number of levels may increase with the increase of the scan distance for the same reason.

Without lateral movement, the level behavior of adhesion force can be observed since the contact area jumps unpredictably from one to another due to the non-linearity mentioned above. With lateral movement and a small scan distance, the situation is almost the same due to the random nature of the pull-off process. Therefore, the level behaviors with and without lateral movement are almost the same.

For some of the experimental results reported above, the force differences between two adjacent levels are nearly equal in magnitude. The charge around all the higher asperities may reach saturation through repeated contact or several back-and-forth scans. Therefore, the amount of charge for higher asperities may also be nearly equal. In this way, the number of asperities inside a specific contact area is related to a certain level. If the number of asperities increases by one more, the force tends to jump to an adjacent upper level and vice versa. As shown in Figure 12b, if the contact area shifts from Circle B to C, the adhesion force will increase by a force difference.

For the experimental outcomes, level behavior is observed with small scan distances for all lateral velocities. That is, lateral velocity has little influence on level behavior. It seems that the behavior of the adhesion force is only dependent on the set of asperities on Au film when the microsphere jumps off. However, the magnitude of the adhesion force is influenced by lateral velocity, and the adhesion force decreases with lateral velocity (Figure 9b). This should be attributed to the breakage of adhesive asperity junctions between two surfaces with lateral movement [13].

In the above discussion, the contributions of lower asperities are neglected for convenience of statement. However, lower asperities may make some contributions to adhesion force. For one thing, lower asperities may be very close to the surface of the microsphere due to the deformation of higher asperities. For another, there may be some charges on the lower asperities due to attached charged wear debris by material wear and the electrical conductivity of the Au surface on the Au film sample. After taking the contributions of lower asperities into account, the above discussion still remains reasonable.

When the scan distance is large, level behavior is not observed, and adhesion forces are randomly distributed. When the scan distance is large, there are lots of asperities on the scanning path. The random nature of a pull-off process means there may be many contact areas on the sample. The number of higher asperities in one contact area may be very different to that of another. For example, in Figure 12c, the numbers of higher asperities in Circles E–J are different. Therefore, the adhesion force can be many values, and eventually the level behavior cannot be observed. It should be noted that the data points measured in a large scanning area of a substrate using the force-volume mode of an AFM are usually randomly distributed (mostly Gaussian distribution) [20,41]. This should be attributed to the fact that the tip can touch many different sets of asperities on the substrate. Measurement using the force-volume mode is very similar to the measurement with long scan distances discussed here. 

In the above discussion, the deformation of both the PS microsphere and Au film is neglected. To further deepen understanding of the contact condition, the Johnson-Kendall-Roberts (JKR) model [42] is used to calculate the contact radius between an Au asperity and PS surface. For simplicity, the Au asperity is assumed to be a sphere with a radius of *R*_T_ =100 nm, and the PS surface is flat. Furthermore, the flat PS surface is assumed to be in contact with three asperities, which have the same shape and are under the same normal load. In the model of JKR, the contact radius ($r_{JKR}$) for a single asperity is expressed as [42]:

(3) $$r_{JKR}={\left\{\frac{3R_{T}}{4E^{*}}\left[F_{load}+3\pi R_{T}W_{12}+\sqrt{6\pi R_{T}W_{12}F_{load}+{\left(3\pi R_{T}W_{12}\right)}^{2}}\right]\right\}}^{1/3}$$

where *F*_load_ is the maximum load for a single asperity (~50/3 nN), *W*_12_ is the work of adhesion, and $E^{*}$ is the equivalent elastic modulus. From $F_{adh}^{}=3\pi R_{T}W_{12}/2$, the work of adhesion can be obtained. For a single asperity, the maximum adhesion force is assumed to be 120 nN (the maximum measured force is $F_{adh}^{}$ = 360 nN). The equivalent elastic modulus is:

(4) $$E^{*}={\left[\left(1-\mu_{1}^{2}\right)/E_{1}+\left(1-\mu_{2}^{2}\right)/E_{2}\right]}^{-1}$$

where *E*_1,2_ are Young’s moduli and *μ*_1,2_ are Poisson ratios of PS and Au materials, respectively. *E*_1_ = 5 GPa [43], *E*_2_ = 69.1 GPa [44], *μ*_1_= 0.33 [45], and *μ*_2_= 0.42 [46]. After calculation, $r_{JKR}$ ≈ 19.4 nm. The contact radius is large for a sphere of 100 nm in radius, which may reinforce the effect of contact electrification. It should be noted that the deformation of Au asperity is much larger than that of the PS surface due to a small Young’s modulus (5 GPa). In a word, the above discussion still remains reasonable when the deformation is taken into consideration.

## 5. Conclusions

The adhesion forces were measured consecutively by repeated contact with and without the lateral movement of a sample, using a microsphere in a very dry environment. Without the lateral movement and with a small scan distance, the adhesion forces are grouped into several levels, and the adhesion force jumps frequently between different levels, which is referred to as “level behavior”. The level behavior was ascribed to the small number of asperity sets on the sample surface that can be touched by the microsphere. A level corresponds to a contact area (a set of asperities inside), and the contact area changes from one to another during consecutive measurements. The number of levels sometimes increases with the scan distance. For each level, varied trends were observed: increasing, decreasing and unchanged, which may be connected with the charge density on the surfaces. The lateral velocity has little influence on level behavior, but the force will decrease with lateral velocity. Also, the wear and tear of the microsphere does not appear to influence level behavior but may lead to an increase in force magnitude and result in a small jump with discontinuity and larger fluctuations of the adhesion force on one level. However, with a large scan distance, level behavior is not observed, and adhesion forces are randomly distributed. This was attributed to the fact that there are large sets of asperities on the scanning path with larger scan distance. With a medium scan distance, the level behavior is observed for some data points, and the other are randomly distributed.

## Data Availability

Not applicable.

## Supplementary Materials

The following are available online at https://www.mdpi.com/1996-1944/14/2/370/s1. Supplemental data for this article includes Figure S1: Adhesion force versus sequential measurement number of times, measured on Au film using Probe 2# with a scan rate of 1 Hz and different scan distances: (a) 10 nm, (b) 20 nm, (c) 40 nm, (d) 80 nm, (e) 160 nm, (f) 320 nm, (g) 640 nm, (h) 1 μm, and (i) 2 μm. Two vertical lines are used to mark the segments (100 data points) that are selected to be replotted in the same figure, Figure S2: Adhesion force versus sequential measurement number of times, measured on Au film using Probe 2# with a scan rate of 1 Hz and different scan distances: (a) 10 nm, (b) 20 nm, (c) 40 nm, (d) 80 nm, (e) 160 nm, (f) 320 nm, (g) 640 nm, (h) 1 μm, (i) 2 μm, and (j) 4 μm. Two vertical lines are used to mark the segments (100 data points) that are selected to be replotted in the same figure, Figure S3: Adhesion force versus sequential measurement number of times, measured on Au film with a scan distance of 640 nm and different scan rates: (a) 0.1 Hz, (b) 1 Hz, (c) 2 Hz, (d) 4 Hz, (e) 8 Hz, (f) 16 Hz, (g) 32 μm, (j) 64 Hz, and (i) 100 Hz. Two vertical lines are used to mark the segments (100 data points) that are selected to be replotted in the same figure, Figure S4: Adhesion force versus sequential measurement number of times, measured on Au film with a scan distance of 6.4 μm and different scan rates: (a) 0.001 Hz, (b) 0.01 Hz, (c) 0.1 Hz, (d) 1 Hz, (e) 10 Hz, and (f) 100 Hz. Two vertical lines are used to mark the segments (100 data points) that are selected to be replotted in the same figure, Figure S5: Adhesion force versus sequential measurement number of times, measured on Au film with different scan distances and different scan rates: (a) *d* = 0; (b) *d* = 10 nm, *f*_scan_ = 64 Hz; (c) *d* = 20 nm, *f*_scan_ = 32 Hz; (d) *d* = 40 nm, *f*_scan_ = 16 Hz; (e) *d* = 80 nm, *f*_scan_ = 8 Hz; (f) *d* = 160 nm, *f*_scan_ = 4 Hz; (g) *d* = 320 nm, *f*_scan_ = 2 Hz; (h) *d* = 640 nm, *f*_scan_ = 1 Hz; (i) *d* = 1.28 μm, *f*_scan_ = 0.5 Hz; (j) *d* = 1.92 μm, *f*_scan_ = 0.333 Hz; (k) *d* = 2.56 μm, *f*_scan_ = 0.25 Hz; (l) *d* = 3.2 μm, *f*_scan_ = 0.2 Hz; (m) *d* = 3.84 μm, *f*_scan_ = 0.167 Hz; (n) *d* = 4.48 μm, *f*_scan_ = 0.143 Hz; (o) *d* = 5.12 μm, *f*_scan_ = 0.125 Hz; and (p) *d* = 12.8 μm, *f*_scan_ = 0.05 Hz. For (b-p). The lateral velocities are the same (1.28 μm/s). Two vertical lines are used to mark the segments (100 data points) that are selected to be replotted in the same figure.

## References

[1] Zhao Y.P., Wang L.S., Yu T.X. (2003). Mechanics of adhesion in MEMS—A review. J. Adhes. Sci. Technol..

[2] Rebeiz G., Muldavin J. (2001). RF MEMS switches and switch circuits. IEEE Microw. Mag..

[3] Adams G.G., McGruer N.E. (2010). A Review of Adhesion in an Ohmic Microswitch. J. Adhes. Sci. Technol..

[4] Song Y.-H., Yoon J.-B. (2015). Micro and Nanoelectromechanical Contact Switches for Logic, Memory, and Power Applications. A New Perspective of Cultural DNA.

[5] Yeo C.-D., Lee S.-C., Polycarpou A.A. (2008). Dynamic adhesive force measurements under vertical and horizontal motions of interacting rough surfaces. Rev. Sci. Instruments.

[6] Chen L., Lee H., Guo Z.J., McGruer N.E., Gilbert K.W., Mall S., Leedy K., Adams G.G. (2007). Contact resistance study of noble metals and alloy films using a scanning probe microscope test station. J. Appl. Phys..

[7] Chen L., McGRUER N.E., Adams G.G., Du Y. (2008). Separation modes in microcontacts identified by the rate dependence of the pull-off force. Appl. Phys. Lett..

[8] Kappl M., Butt H.J. (2002). The colloidal probe technique and its application to adhesion force measurements. Part. Part. Syst. Charact..

[9] Nasrallah H., Mazeran P.-E., Noël O. (2011). Circular mode: A new scanning probe microscopy method for investigating surface properties at constant and continuous scanning velocities. Rev. Sci. Instrum..

[10] Noël O., Mazeran P.-E., Nasrallah H. (2012). Sliding Velocity Dependence of Adhesion in a Nanometer-Sized Contact. Phys. Rev. Lett..

[11] Sirghi L. (2012). Transport Mechanisms in Capillary Condensation of Water at a Single-Asperity Nanoscopic Contact. Langmuir.

[12] Mazeran P.-E. (2006). Effect of sliding velocity on capillary condensation and friction force in a nanoscopic contact. Mater. Sci. Eng. C.

[13] Lee J., Maharjan J., He M., Yeo C.-D. (2015). Effects of system dynamics and applied force on adhesion measurement in colloidal probe technique. Int. J. Adhes. Adhes..

[14] Lai T., Meng Y., Zhan W., Huang P. (2017). Logarithmic decrease of adhesion force with lateral dynamic revealed by an AFM cantilever at different humidities. J. Adhes..

[15] Lai T., Meng Y., Tang H., Yu G. (2017). Influence of lateral velocity on adhesion force of surfaces with different hydrophilicity revealed by an AFM colloidal probe at humid environments. J. Adhes..

[16] Çolak A., Wormeester H., Zandvliet H.J.W., Poelsema B. (2014). The influence of instrumental parameters on the adhesion force in a flat-on-flat contact geometry. Appl. Surf. Sci..

[17] Çolak A., Wormeester H., Zandvliet H.J., Poelsema B. (2015). The influence of instrumental parameters on the adhesion force in a flat-on-rough contact geometry. Appl. Surf. Sci..

[18] Ibrahim T.H., Burk T.R., Neuman R.D., Etzler F.M. (2000). Direct adhesion measurements of pharmaceutical particles to gelatin capsule surfaces. J. Adhes. Sci. Technol..

[19] Tormoen G.W., Drelich J. (2005). Deformation of soft colloidal probes during AFM pull-off force measurements: Elimination of nano-roughness effects. J. Adhes. Sci. Technol..

[20] Lai T., Huang P. (2013). Study on microscale adhesion between solid surfaces with scanning probe. Sci. China Ser. E Technol. Sci..

[21] Lai T., Zhu S., Huang P. (2016). Adhesion Behavior of a Grating at a Single Location by Using an AFM Flat Tip Under Different Conditions. J. Adhes..

[22] Lai T., Meng Y. (2017). Behaviors of time-dependent and time-independent adhesion forces revealed using an AFM under different humidities and measuremental protocols. Int. J. Adhes. Adhes..

[23] Lai T., Meng Y. (2017). Logarithmic contact time dependence of adhesion force and its dominant role among the effects of AFM experimental parameters under low humidity. Appl. Surf. Sci..

[24] Lai T., Meng Y., Yang Q., Huang P. (2017). Evolution and level behavior of adhesion force by repeated contacts of an AFM colloid probe in dry environment. J. Adhes..

[25] Hutter J.L., Bechhoefer J. (1993). Calibration of atomic-force microscope tips. Rev. Sci. Instrum..

[26] Butt H.-J., Cappella B., Kappl M. (2005). Force measurements with the atomic force microscope: Technique, interpretation and applications. Surf. Sci. Rep..

[27] Lacks D.J., Sankaran R.M. (2011). Contact electrification of insulating materials. J. Phys. D Appl. Phys..

[28] Izadi H., Penlidis A. (2013). Polymeric Bio-Inspired Dry Adhesives: Van der Waals or Electrostatic Interactions?. Macromol. React. Eng..

[29] Henniker J.C. (1962). Triboelectricity in Polymers. Nat. Cell Biol..

[30] Diaz A., Felix-Navarro R. (2004). A semi-quantitative tribo-electric series for polymeric materials: The influence of chemical structure and properties. J. Electrost..

[31] Gooding D.M., Kaufman G.K. (2014). Tribocharging and the Triboelectric Series. Encyclopedia of Inorganic and Bioinorganic Chemistry.

[32] Terris B.D., Stern J.E., Rugar D., Mamin H.J. (1989). Contact electrification using force microscopy. Phys. Rev. Lett..

[33] Baytekin H.T., Patashinski A.Z., Branicki M., Baytekin B., Soh S., Grzybowski B.A. (2011). The Mosaic of Surface Charge in Contact Electrification. Science.

[34] Horn R.G., Smith D.T. (1992). Contact Electrification and Adhesion between Dissimilar Materials. Science.

[35] McGuiggan P.M. (2008). Stick Slip Contact Mechanics between Dissimilar Materials: Effect of Charging and Large Friction. Langmuir.

[36] Izadi H., Zhao B., Han Y., McManus N., Penlidis A. (2012). Teflon hierarchical nanopillars with dry and wet adhesive properties. J. Polym. Sci. Part B Polym. Phys..

[37] Watson P.K., Yu Z.-Z. (1997). The Contact Electrification of Polymers and the Depth of Charge Penetration. J. Electrost..

[38] Guerret-Piecourt C., Bec S., Treheux D. (2001). Electrical charges and tribology of insulating materials. Comptes Rendus de l’Académie des Sciences Series IV Physics.

[39] Castle G.S.P., Schein L. (1995). General model of sphere-sphere insulator contact electrification. J. Electrost..

[40] Baytekin H.T., Baytekin B., Incorvati J.T., Grzybowski B.A. (2012). Material Transfer and Polarity Reversal in Contact Charging. Angew. Chem..

[41] Lai T., Chen R., Huang P. (2015). Temperature dependence of microscale adhesion force between solid surfaces using an AFM. J. Adhes. Sci. Technol..

[42] Johnson K.L., Kendall K., Roberts A.D. (1971). Surface energy and the contact of elastic solids. Proc. R. Soc. London. Ser. A. Math. Phys. Sci..

[43] Guo D., Li J., Xie G., Wang Y., Luo J. (2014). Elastic Properties of Polystyrene Nanospheres Evaluated with Atomic Force Microscopy: Size Effect and Error Analysis. Langmuir.

[44] Salvadori M.C., Vaz A.R., Melo L.L., Cattani M. (2003). Nanostructured Gold Thin Films: Young Modulus Measurement. Surf. Rev. Lett..

[45] Tan S., Sherman R.L., Ford W.T. (2004). Nanoscale Compression of Polymer Microspheres by Atomic Force Microscopy. Langmuir.

[46] Samsonov G.V. (1968). Handbook of the Physicochemical Properties of the Elements.

