# Supplementary material for: Influence of Lateral Movement on Level Behavior of Adhesion Force Measured Repeatedly by an Atomic Force Microscope (AFM) Colloid Probe in Dry Conditions
Source: Materials (Basel). 2021 Jan 13;14(2):370. doi: 10.3390/ma14020370 (PMC7828610; doi:10.3390/ma14020370)
Supplement: Supplementary file 1 [file materials-14-00370-s001.pdf]

# Supplementary Materials for

# Influence of Lateral Movement on Level Behavior of Adhesion Force Measured Repeatedly by an AFM Colloid Probe in Dry Conditions

Ping Li and Tianmao Lai *

*School of Mechanical and Electric Engineering, Guangzhou University, Guangzhou 510006, China*

*Corresponding author: Email: laitianmao@163.com

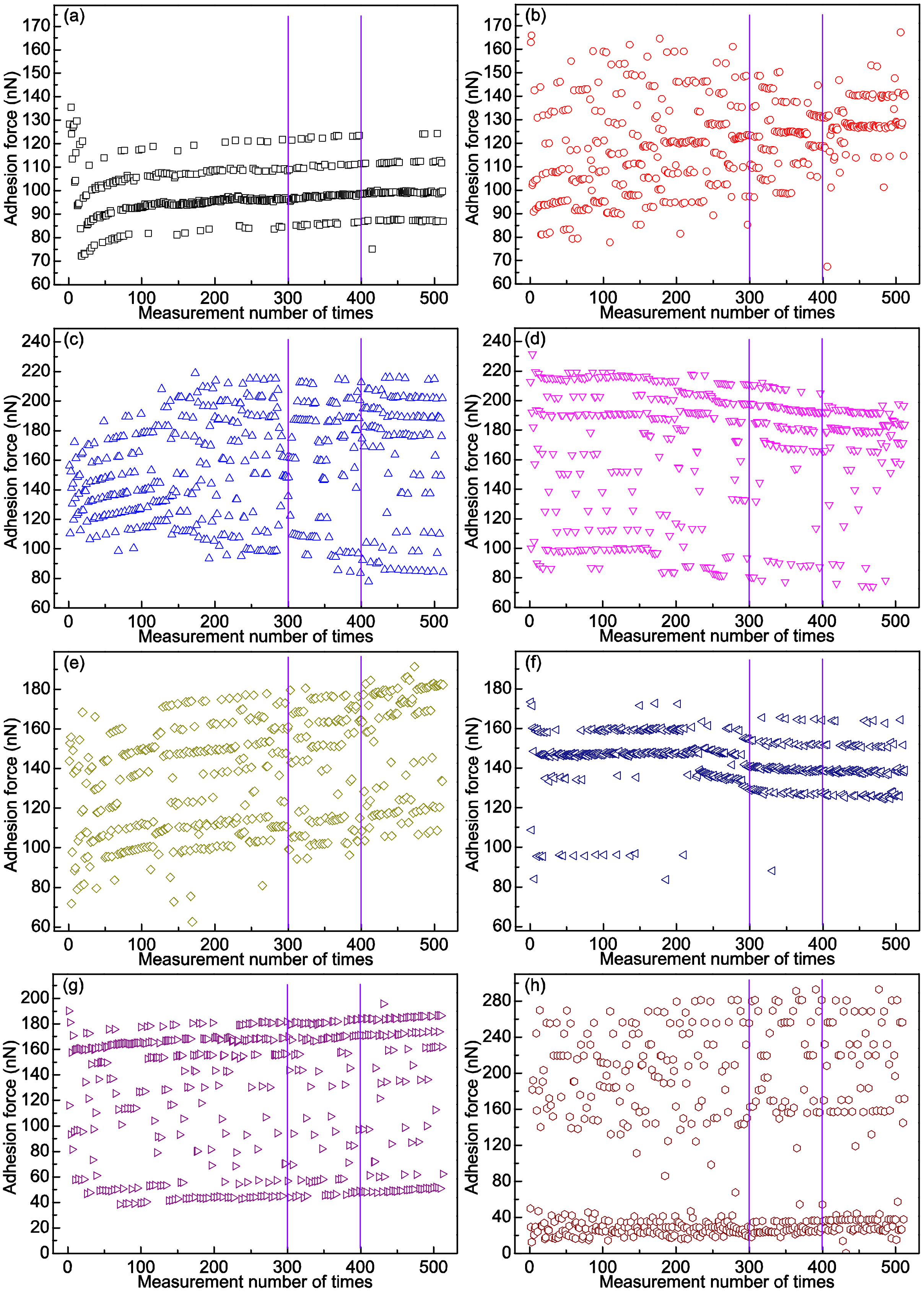


Figure S1. Adhesion force versus sequential measurement number of times, measured on Au film using Probe 2# with a scan rate of 1 Hz and different scan distances: (a) 10 nm, (b) 20 nm, (c) 40 nm, (d) 80 nm, (e) 160 nm, (f) 320 nm, (g) 640 nm, (h) 1 μm, and (i) 2 μm. Two vertical lines are used to mark the segments (100 data points) that are selected to be replotted in the same figure.

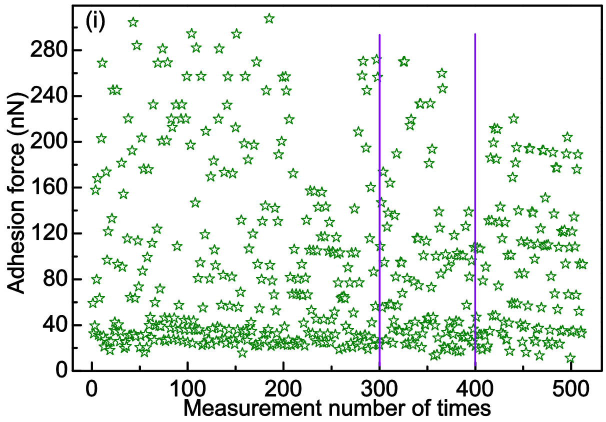


Figure S1. (Continued)

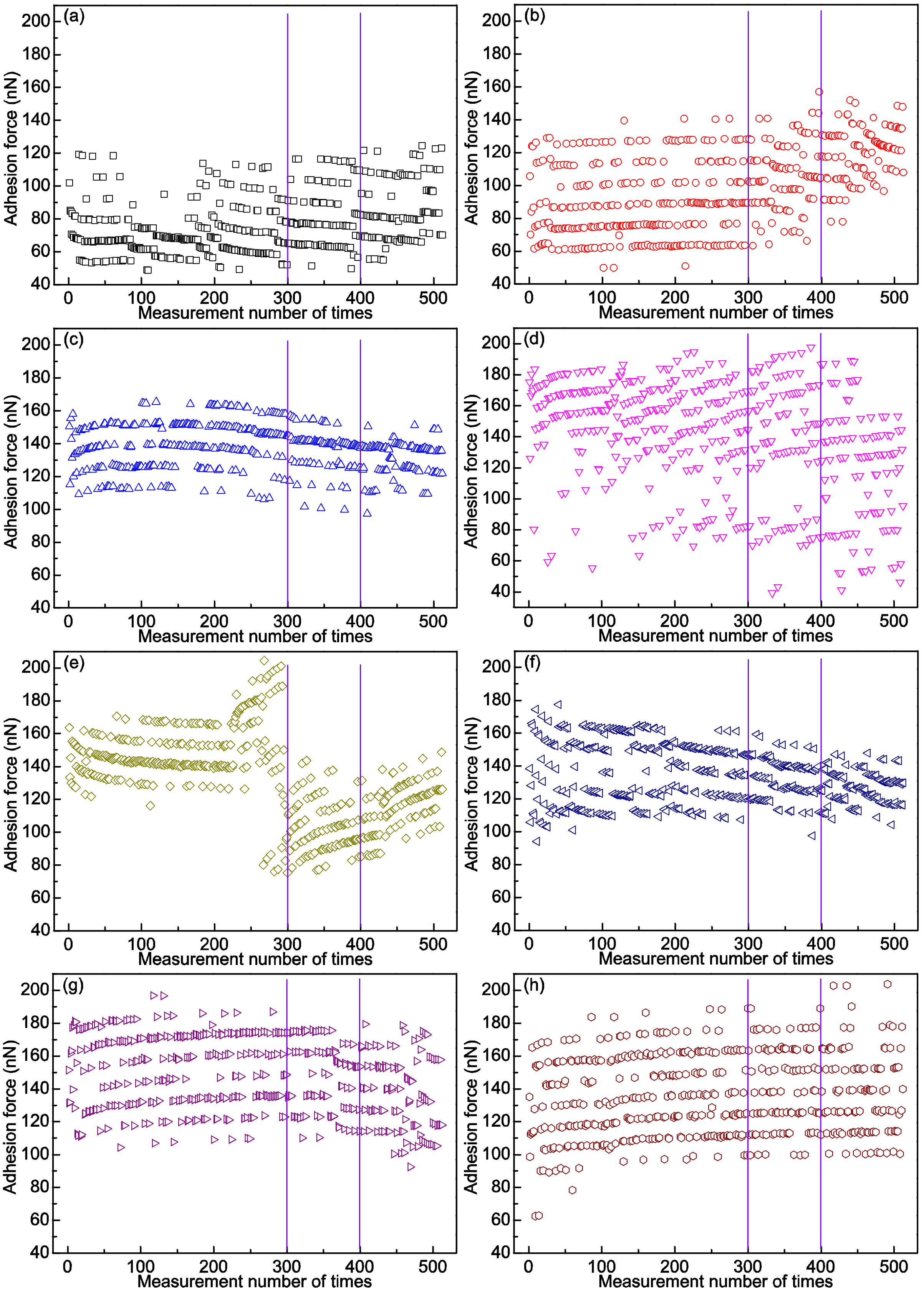


Figure S2. Adhesion force versus sequential measurement number of times, measured on Au film using Probe 2# with a scan rate of 1 Hz and different scan distances: (a) 10 nm, (b) 20 nm, (c) 40 nm, (d) 80 nm, (e) 160 nm, (f) 320 nm, (g) 640 nm, (h) 1 μm, (i) 2 μm, and (j) 4 μm. Two vertical lines are used to mark the segments (100 data points) that are selected to be replotted in the same figure.

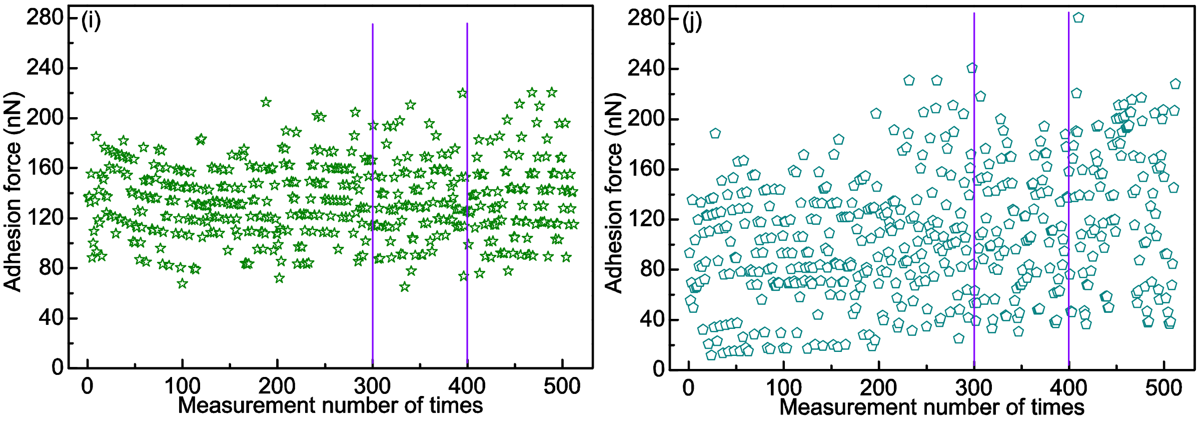


Figure S2. (Continued)

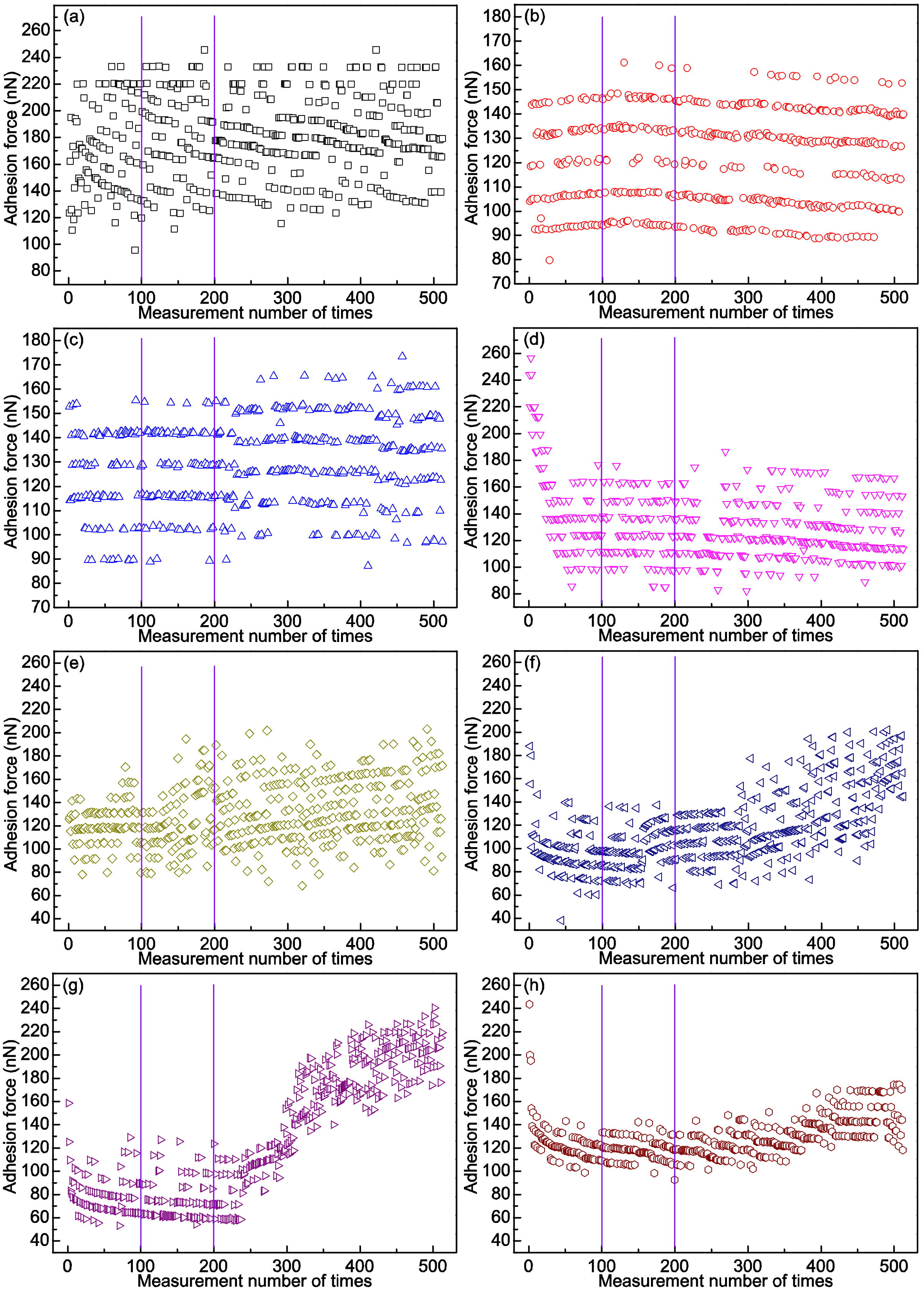


Figure S3. Adhesion force versus sequential measurement number of times, measured on Au film with a scan distance of 640 nm and different scan rates: (a) 0.1 Hz, (b) 1 Hz, (c) 2 Hz, (d) 4 Hz, (e) 8 Hz, (f) 16 Hz, (g) 32 μm, (j) 64 Hz, and (i) 100 Hz. Two vertical lines are used to mark the segments (100 data points) that are selected to be replotted in the same figure.

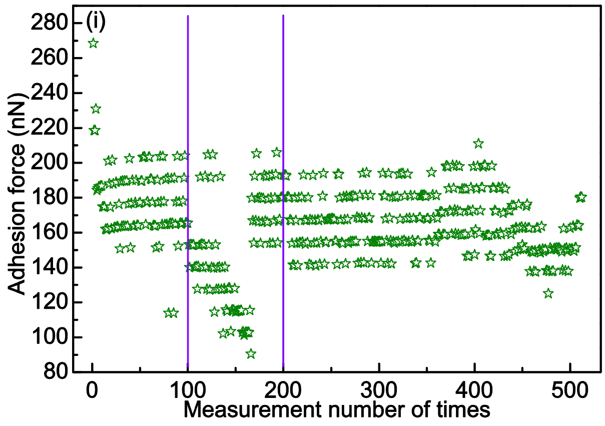


Figure S3. (Continued)

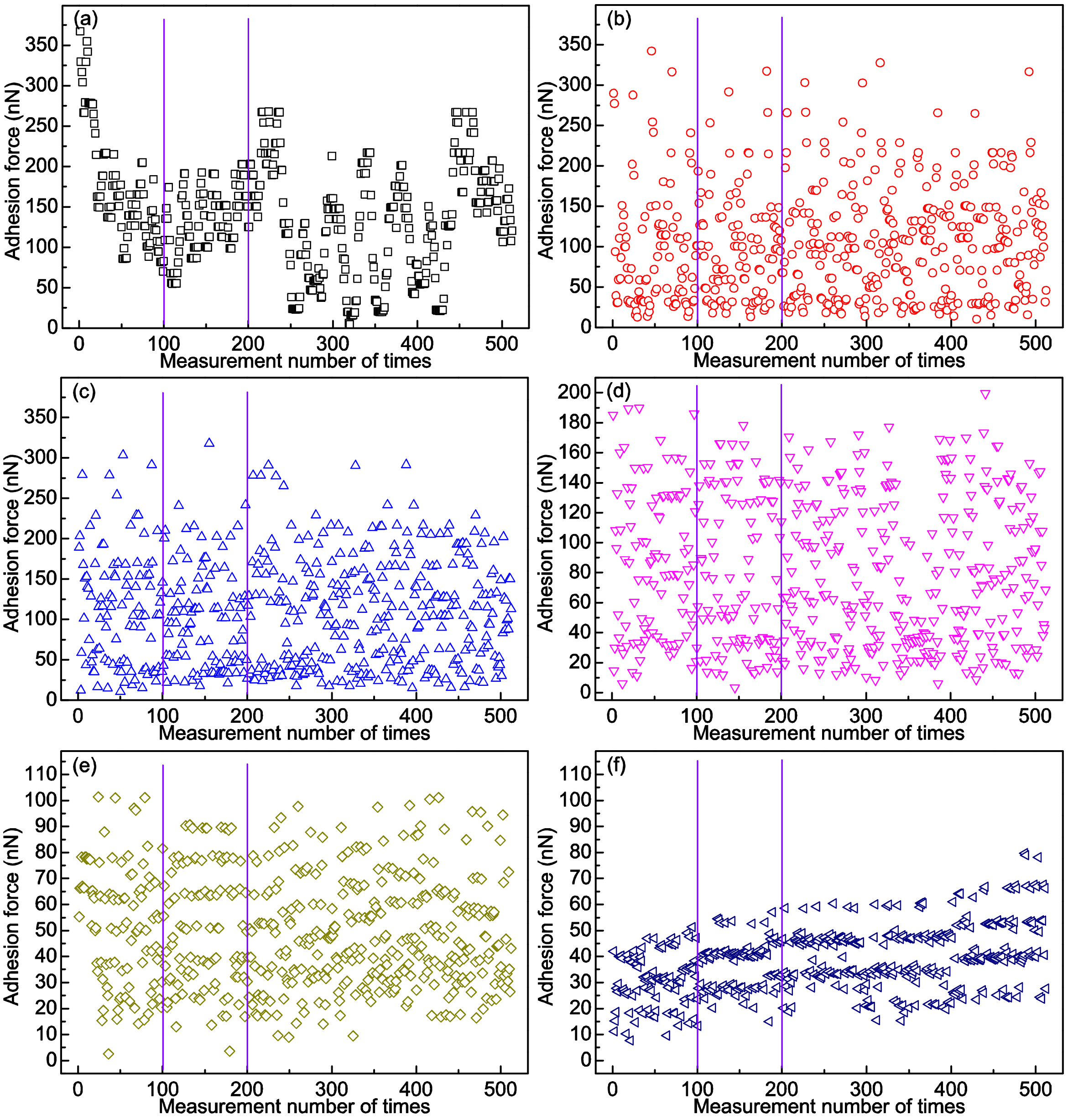


Figure S4. Adhesion force versus sequential measurement number of times, measured on Au film with a scan distance of 6.4 μm and different scan rates: (a) 0.001 Hz, (b) 0.01 Hz, (c) 0.1 Hz, (d) 1 Hz, (e) 10 Hz, and (f) 100 Hz. Two vertical lines are used to mark the segments (100 data points) that are selected to be replotted in the same figure.

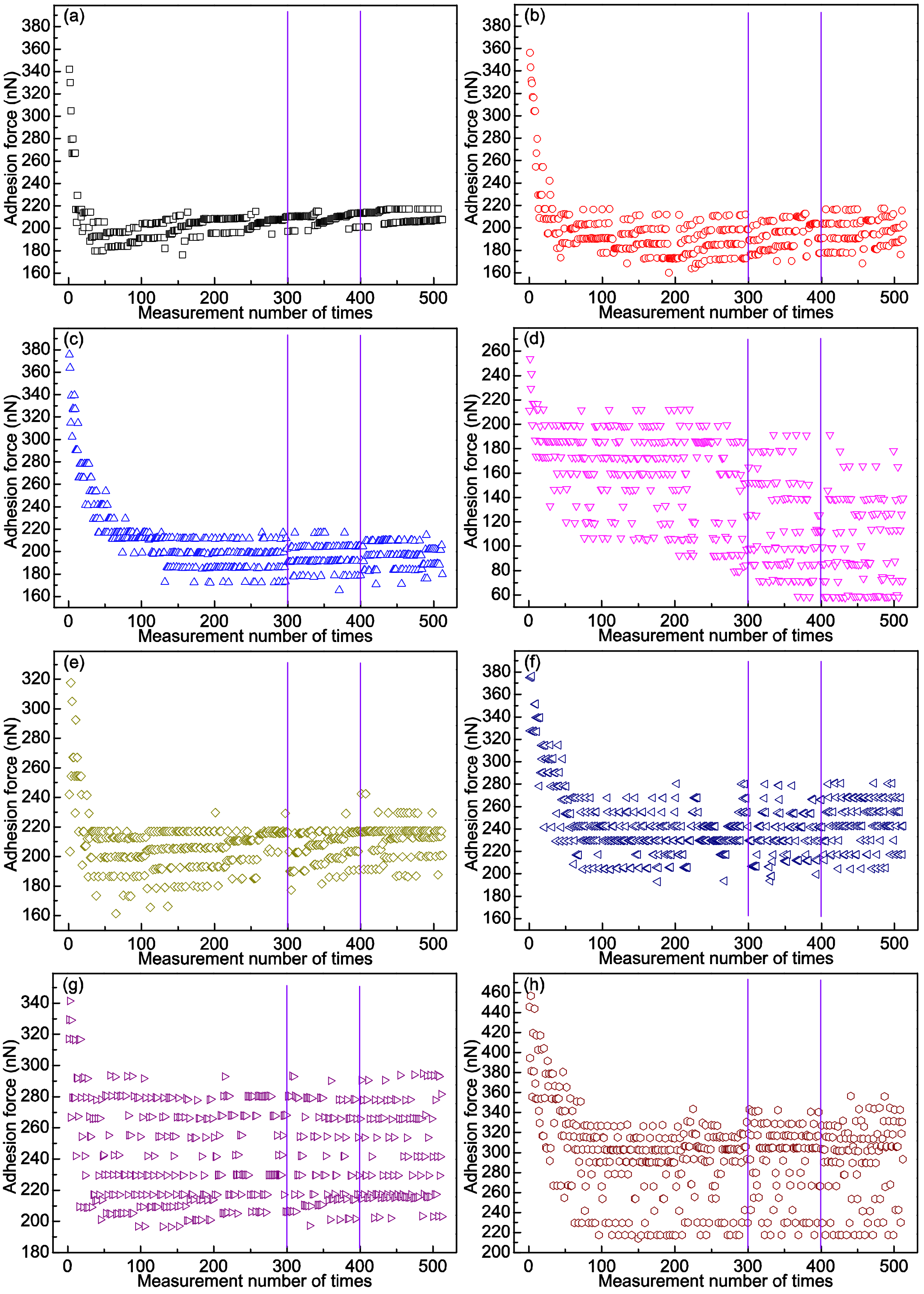


Figure S5. Adhesion force versus sequential measurement number of times, measured on Au film with different scan distances and different scan rates: (a) $d$ = 0; (b) $d$ = 10 nm, $f_{scan}$ = 64 Hz; (c) $d$ = 20 nm, $f_{scan}$ = 32 Hz; (d) $d$ = 40 nm, $f_{scan}$ = 16 Hz; (e) $d$ = 80 nm, $f_{scan}$ = 8 Hz; (f) $d$ = 160 nm, $f_{scan}$ = 4 Hz; (g) $d$ = 320 nm, $f_{scan}$ = 2 Hz; (h) $d$ = 640 nm, $f_{scan}$ = 1 Hz; (i) $d$ = 1.28 μm, $f_{scan}$ = 0.5 Hz; (j) $d$ = 1.92 μm, $f_{scan}$ = 0.333 Hz; (k) $d$ = 2.56 μm, $f_{scan}$ = 0.25 Hz; (l) $d$ =

3.2 μm, $f_{scan}$ = 0.2 Hz; (m) $d$ = 3.84 μm, $f_{scan}$ = 0.167 Hz; (n) $d$ = 4.48 μm, $f_{scan}$ = 0.143 Hz; (o) $d$ = 5.12 μm, $f_{scan}$ = 0.125 Hz; and (p) $d$ = 12.8 μm, $f_{scan}$ = 0.05 Hz. For (b-p). The lateral velocities are the same (1.28 μm/s). Two vertical lines are used to mark the segments (100 data points) that are selected to be replotted in the same figure.

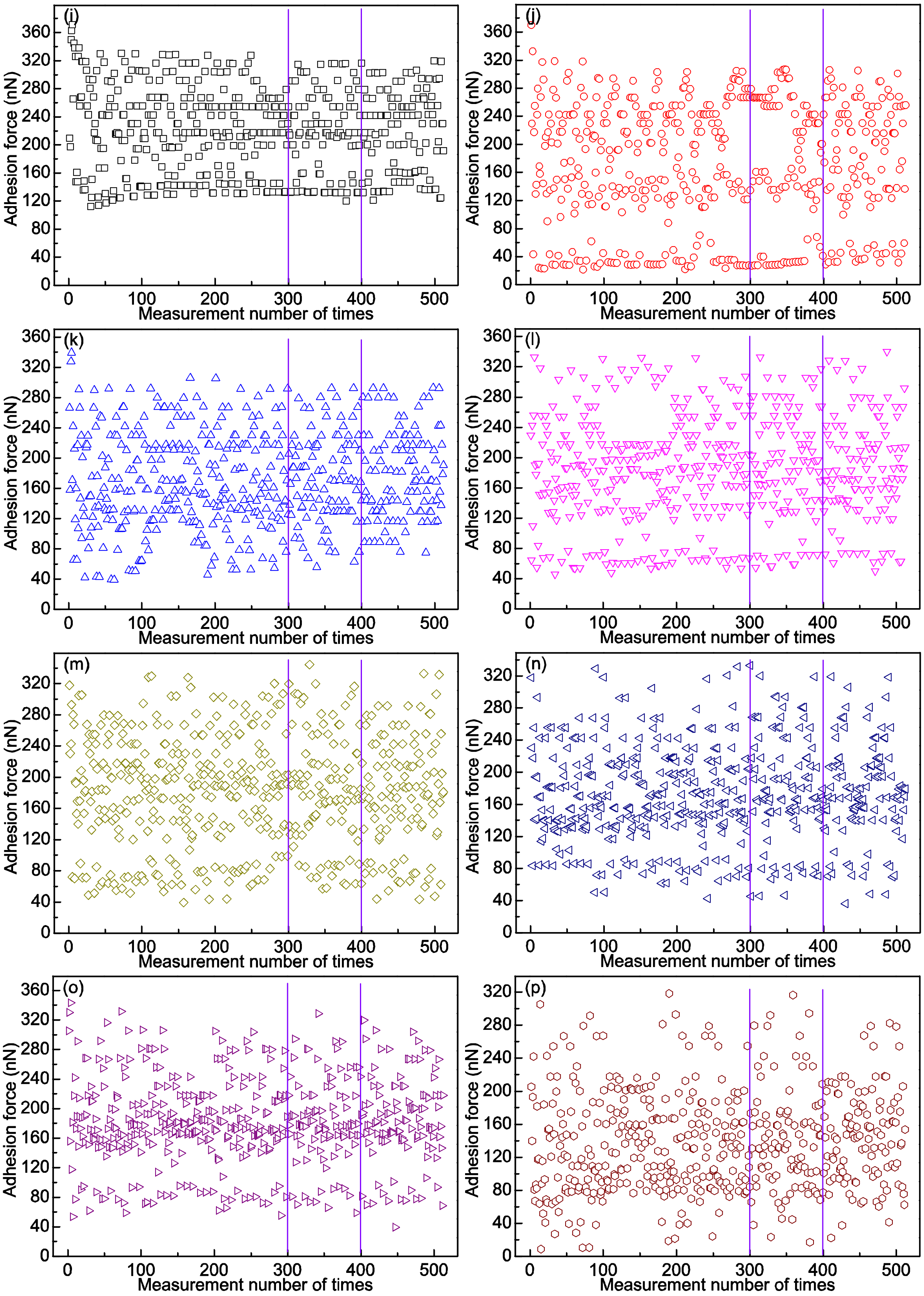


Figure S5. (Continued)
